# Male *Pagurus minutus* hermit crabs use multiple types of information in decisions to give up male–male contests

**DOI:** 10.1038/s41598-023-47947-3

**Published:** 2023-11-24

**Authors:** Chiaki I. Yasuda, Tsunenori Koga

**Affiliations:** 1 Faculty of Education, Wakayama University, Sakaedani, Wakayama, 640-8510 Japan; 2 Graduate School of Fisheries Sciences, Hokkaido University, Minato-cho, Hakodate, Hokkaido 041-8611 Japan

**Keywords:** Behavioural ecology, Animal behaviour

## Abstract

Organisms use information to make adaptive decisions in various contexts, including aggression. Potentially weaker, but better-informed, contestants should give up earlier to reduce fighting costs by using information related to their own lower success such as their size relative to their opponent and past contest outcomes to make this choice. Here, we examined whether intruders of the hermit crab *Pagurus minutus* could use information about their (1) smaller size, (2) past contest defeats, (3) opponent’s past wins, or (4) relationship in the dominance hierarchy to their opponent when making a decision to give up during male–male contests for a female. In all trials, we randomly matched a smaller intruder with a larger opponent that was guarding a female. Our analyses suggest that *P. minutus* intruders can use all four types of information to decide whether to give up a contest without escalation or decrease its duration after escalation; it is the first species of *Pagurus* reported to do so, and the second reported to be able to distinguish familiar opponents from others in the context of male–male contests. These findings demonstrate the importance of cognitive abilities in minimizing costs when competing for vital resources.

## Introduction

Information is crucial for organisms making adaptive decisions in various contexts. Better-informed individuals can adjust their behavior to meet demands^1^, and many species have information about both their current condition and past experience^2–4^. For example, juveniles of the perch *Perca fluviatilis* decrease their feeding rate in response to a visual cue of a predator and increase the intensity of their antipredator response when they have both visual and olfactory cues^5^. Males of the wolf spider *Schizocosa ocreata* can modify their courtship display according to current and past conditions, such as the number of courting male stimuli, the presence of female cues, and field experience^6^. Females of the Japanese pygmy squid *Idiosepius paradoxus* tend to abstain from attacking larger prey after learning that large prey are less likely to be captured^7^.

Contests for limited resources involve information use. Since contest outcomes are strongly affected by asymmetry of fighting ability or resource-holding potential (RHP)^8^ between contestants, information related to RHP asymmetry is key to the decision to give up or persist in contests^9,10^. Since weaker contestants often incur a greater cost than stronger contestants^11,12^, contestants that are potentially weaker but better informed should give up earlier to reduce costs on the basis of their assessment of their own lower RHP and/or relatively lower RHP to opponents’. Body size is a common information source used to assess RHP, and smaller contestants give up sooner^13^. Past contest outcomes and previously established dominance hierarchy also affect giving-up decisions in weaker contestants^14–17^. These experiences might provide information for re-estimation of their own RHP (i.e., self-assessment)^18,19^, as an additional source of information for assessment of their opponent’s RHP (i.e., social cue)^20^, and as a reliable source of information for assessment of RHP asymmetry against a particular opponent (i.e., familiar recognition)^21^.

Males of hermit crabs in the genus *Pagurus* have direct contests for mates during precopulatory guarding^22,23^, in which the male grasps the aperture of the gastropod shell occupied by a sexually mature female over several days^24,25^. The male–male contests are initiated by physical aggression of solitary intruders^22,23^ and are often settled in favor of the larger male^22,26,27^. Smaller males are therefore potentially weaker in this context. To decide when to give up the contest, when they encounter larger guarding opponents, smaller intruders might gather information about their chance of success, such as their relative size, their own recent defeats or their opponent’s recent wins, and the dominance hierarchy with their opponent.

Yasuda et al.^28^ examined whether these four factors affect the decision of *P. middendorffii* intruders to give up by using pairs of randomly selected smaller intruders and larger guarding males (i.e., random-sized method^19^). This species shows a large size advantage in male–male contests^26^, and smaller intruders are less likely to escalate fights^23^. This avoidance increased when smaller intruders encountered unfamiliar larger opponents with previous experience of wins, and avoidance was greatest when they re-encountered familiar opponents that had established dominance hierarchy^28^. Their own experience of defeat, however, did not affect the decision to give up in this species^28^. Thus, smaller intruders of *P. middendorffii* appear to use three types of information for their decision, but not their own defeats.

*Pagurus minutus* is another species in which the effects of information on the intruder’s decision to give up has been investigated. *Pagurus minutus* also shows a large size advantage and a lower probability of escalation by smaller intruders^29,30^. Yasuda et al.^31^ reported that *P. minutus* intruders with previous defeats had decreased eventual fighting success against unfamiliar naïve guarders. Although this suggests that individuals of this species collect information about experience of defeat, the effect of this information on the decision to give up is still unclear, because the study did not assess whether and when the losers gave up. More importantly, it used pairs of similar-sized males (i.e., self-selection method^19^) to clarify the effect of factors other than male size (i.e., female size in that study) on male–male contests. Hsu et al.^19^ recommend the random-sized method to examine the effect of experience, because the relationship between size and RHP is not perfect, even between similar-sized contestants. No study has tested whether *P. minutus* can use information related to opponent status and established hierarchy.

Here, we examined whether the decision of *P. minutus* intruders to give up is affected by four types of potentially available information related to RHP, namely (1) size relative to their opponent, (2) previous defeats, (3) opponent’s previous wins, and (4) established dominance hierarchy with the same opponent. We used randomly-selected males in male–male contests according to the suggestion of Hsu et al.^19^ (also see Yasuda et al.^31^).

## Methods

### Study animals

We collected precopulatory guarding pairs of *P. minutus*, each male with an intact major cheliped, from a sandy mud flat at Nunohiki, in the Waka River estuary, Wakayama, Japan (34°10′23″N, 135°10′49″E), from December 2015 to February 2016; the mating season of this species at this site occurs from November to April^32^. Each pair was placed in a small vinyl pouch filled with seawater collected in the field. In the laboratory, pairs in which the male was still guarding the female were separated, and each individual was kept in a container (8 cm × 12.5 cm × 8 cm) or a plastic cup (200 mL) with natural seawater (2.5 cm deep), to prevent copulation before the experiment. All pairs were acclimatized to laboratory conditions for at least 1 h before the experiment, and all tests were conducted within 6 h of collection.

After the experiments, all crabs were fixed by freezing (− 18°C) to allow us to measure them. The shield length (SL, calcified anterior portion of the cephalothorax, index of body size) of all males was then measured to the nearest 0.01 mm under a stereomicroscope. Since female size has no effect on random-sized male–male contests in this species^29,30^, we excluded this value.

### Experimental design

We performed two sequential trials of male–male contests (Trials 1 and 2). In Trial 1, two guarding pairs were randomly assigned to an experimental set (*N* = 92 sets), and in each set the smaller male was designated as the intruder and the larger male as the guarder, owing to the large size advantage in this species^29^. We then placed a guarding male and his guarded female in a small plastic arena (19.5 cm × 11.0 cm × 8.5 cm) containing seawater about 3 cm deep. After the guarder had returned to guarding the female, the intruder was placed in the arena. We checked the outcome of Trial 1 at 15 min from when the intruder initiated movement; all intruders lost Trial 1 (i.e., did not guard a contested female).

Each intruder was then used again as an intruder in a second trial (Trial 2) after 1 h had elapsed. In Trial 2, we assigned the losers to three experimental groups with different types of guarders. In Group 1, losers encountered larger guarders that had not participated in Trial 1 (*N* = 31 sets). In Group 2, losers encountered guarders that won Trial 1 against a different intruder (*N* = 30 sets). In Group 3, losers encountered the same guarders as in Trial 1 (*N* = 31 sets). The difference in SL between losers and guarders did not differ significantly among groups (ANOVA, *F*_2,89_ = 1.030, *P* = 0.361). Other experimental methods in Trial 2 were the same as in Trial 1.

We recorded all trials using a digital camera (DMC-LF1, Panasonic) from the time the individuals were introduced into the arena until 15 min after the intruder began moving. When the intruder initiated grappling with the guarder (for details of this behavior, see^23^), we considered that the trial had escalated. After escalation, if intruders did not perform physical aggression for more than 3 min, we defined the fight as settled. We then recorded the duration (seconds) of the series of aggressive interactions as the contest duration until the intruder gave up and the eventual outcome on the basis of which male was guarding the female. Because the duration was defined as ending when the intruder gave up, we excluded contests in which the intruder won (Group 1, *N* = 1 set; Group 2, *N* = 1 set; Table 1) from the following analyses. If males continued grappling, with both males grabbing the shell of the contested female, at the end of Trial 2, the trial was defined as a draw (Group 1, *N* = 1 set; Table 1), and the duration until giving up the trial was censored. The final sample sizes for the analyses were 30 in Group 1, 29 in Group 2, and 31 in Group 3 (Table 1). No crabs were injured or lost any appendages during either trial.

**Table 1:** Experimental groups for two sequential trials of male–male contests in *Pagurus* *minutus*.

Trial	Exp. group	Status of intruders and guarders (all sets consisted of a smaller intruder and a larger guarder)	*N* (sets)	Intruders’ contest choices			
				Giving-up without escalation	Eventual outcome for intruder		
					Win	Draw	Loss
1	–	Intruders and guarders had no trial experience	92^*1,*2^	20	0	0	92
2	1	Intruder lost Trial 1 vs. guarder with no trial experience	31	16	1^*2^	1	29
	2	Intruder lost Trial 1 vs. guarder that won against a different intruder	30	14	1^*2^	0	29
	3	Intruder lost Trial 1 vs. the same guarder as in Trial 1	31	24	0	0	31

^*1^Data from Trial 1 were divided for analyses focusing on each group (see text).

^*2^These data were excluded from the analysis since our aim was to examine intruders’ giving-up decisions; *N* = 90 for Trial 1 in analyses.

Since the contest duration of Trial 1 had no effect on the decision to give up without escalation in Trial 2 in Group 1 (see Supplementary Fig. S1), we considered that the loser’s behavior in Trial 2 was independent of energy depletion from Trial 1.

### Analyses

Data from Trial 1 were used to examine whether the intruder’s decision to give up was affected by the opponent’s relative size. For giving-up without escalation, a generalized linear model (GLM) with a binomial error distribution was used. This model was constructed by using whether intruders gave up without escalation (Yes = 1, No = 0; *N* = 90) as the response variable and the SL difference between intruders and guarders (DSL_I–G_) as the explanatory variable. Contest duration until giving-up was analyzed by Cox’s proportional hazard model^33^. The response variable in this model was contest duration (sec, *N* = 70), and the explanatory variable was DSL_I–G_.

We then used data from both trials in Group 1 to examine the effect of a defeat in Trial 1 on the decision in Trial 2. Since we observed all intruders twice, we used a generalized linear mixed model (GLMM) and Cox’s model with mixed effects to control for pseudo-replication. In the GLMM, the response variable was whether intruders gave up without escalation (Yes = 1, No = 0; *N* = 30 × 2 = 60), and the explanatory variables were (1) Trial (Trial 1 or 2) and (2) DSL_I–G_. In the mixed Cox’s model, the response variable was contest duration (sec; *N* = 23 + 14 = 37), and the explanatory variable was the same as in the GLMM (i.e., (1) and (2)). Intruder ID was treated as a random factor in both analyses.

The GLMM for giving-up frequency and the mixed Cox’s model for contest duration were also used to assess whether the intruder’s decision was affected by the opponent’s previous wins (Group 1 vs. Group 2; losers faced naïve opponents or opponents that won the previous contest) and established hierarchy with the same opponent (Group 2 vs. Group 3; losers faced a different or the same opponent that won the previous contest). In the GLMMs (Group 1 vs. 2, *N* = [30 × 2] + [29 × 2] = 118; Group 2 vs. 3, *N* = [29 × 2] + [31 × 2] = 120), the explanatory variables were (1) Trial (Trial 1 or 2), (2) Group (Group 1 vs. Group 2 or Group 2 vs. Group 3), and (3) DSL_I–G_. In the mixed Cox’s model (Group 1 vs. Group 2, *N* = 23 + 14 + 22 + 15 = 74; Group 2 vs. Group 3, *N* = 22 + 15 + 25 + 7 = 69), all three explanatory variables were included. To examine the effect of information, we also added a (4) Trial × Group interaction in each model if the interaction was significant. Intruder ID was treated as a random effect in all four models.

All analyses in this study were performed in R v. 4.1.1^34^ software, and the R packages “glmmML”^35^ and “coxme”^36^ were used to conduct the GLMM and Cox’s model with mixed effects analyses. In Cox’s model, the proportional hazard assumption was satisfied for all explanatory variables (*P* > 0.062), except for a Trial × Group interaction in the model comparing Groups 2 and 3 (*P* = 0.034). However, since this model was not used in this study because the Trial × Group interaction was not significant (see “Results”), we concluded the proportional hazard assumption to be acceptable.

## Results

### Relative size

**Figure 1:**
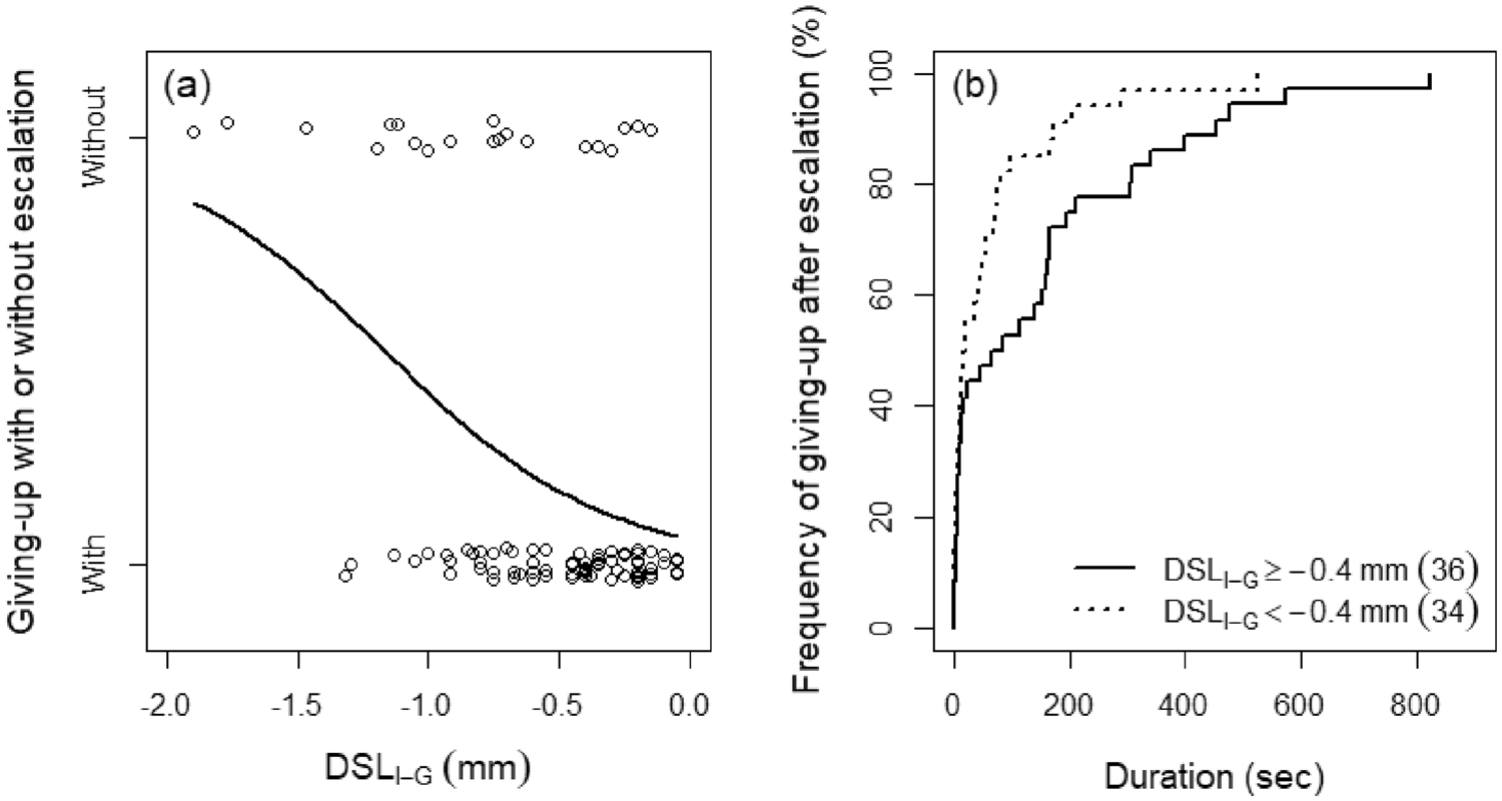
Relationship between male size difference and (**a**) intruders’ frequency of giving-up without escalation and (**b**) contest duration after escalation. DSL_I–G_ indicates difference in shield length (index of body size) between intruders and guarders. Number in parentheses in (**b**) indicates sample size.

In Trial 1, the frequency of giving-up without escalation significantly increased as DSL_I–G_ decreased (*z* = − 3.234, *P* = 0.001; Fig. 1a). After escalation, smaller intruders also showed significantly earlier giving-up (*z* = − 3.435, *P* < 0.001; Fig. 1b). Details of these and following analyses are shown in Supplementary Table S1.

### Prior defeat

**Figure 2:**
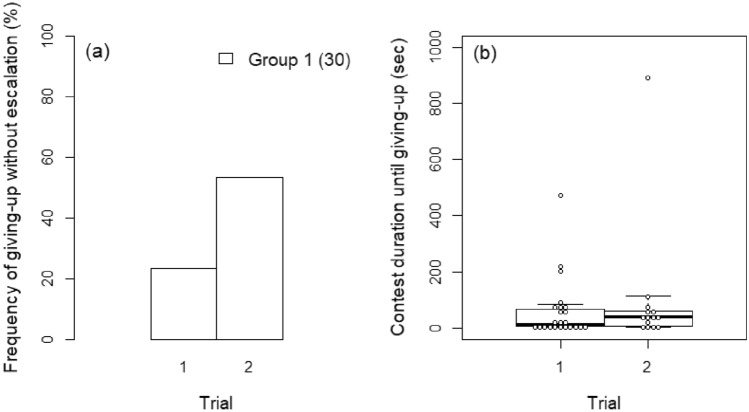
Relationship between trials and (**a**) the frequency of giving-up without escalation by intruders and (**b**) duration until giving-up by intruders after escalation. Number in parentheses in (**a**) indicates sample size in Group 1.

In Group 1, the frequency of giving-up without escalation was significantly higher in Trial 2 (after defeat) than in Trial 1 (before defeat) (*z* = 2.264, *P* = 0.024; Fig. 2a), but was not affected by DSL_I–G_ (*z* = − 1.687, *P* = 0.092). Contest duration in this group was independent of both trial number and DSL_I–G_ (Trial: *z* = − 0.560, *P* = 0.576; DSL_I–G_: *z* = − 1.302, *P* = 0.193; Fig. 2a).

### Opponent’s prior wins

**Figure 3:**
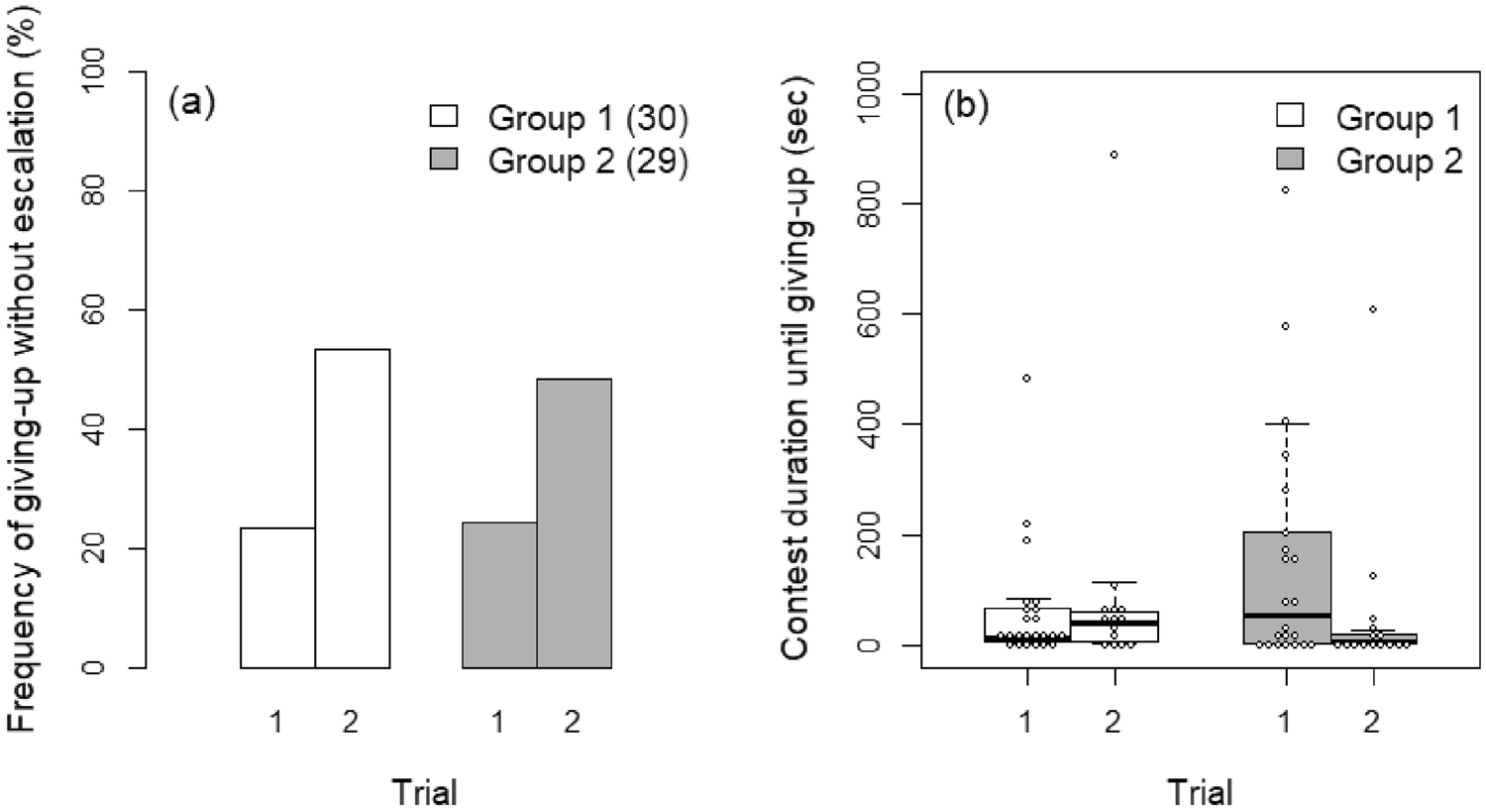
Differences between Groups 1 and 2 in (**a**) the frequency of giving-up without escalation by intruders and (**b**) duration until giving-up by intruders after escalation. Numbers in parentheses in (**a**) indicate sample size. Group 1: naïve opponent; Group 2: opponent that won against a different intruder.

In the comparison between Groups 1 and 2, Trial × Group interaction was excluded from the GLMM because it was not significant (z = − 0.562, *P* = 0.574). In the GLMM without the interaction, giving-up without escalation was significantly affected by Trial and DSL_I–G_ but not by Group (Trial: *z* = 2.925, *P* = 0.003; DSL_I–G_: *z* = − 2.512, *P* = 0.012; Group: *z* = − 0.412, *P* = 0.680). The frequency of intruders that did not escalate was higher in Trial 2 than in Trial 1 of both groups (Fig. 3a) and increased as DSL_I–G_ decreased.

**Figure 4:**
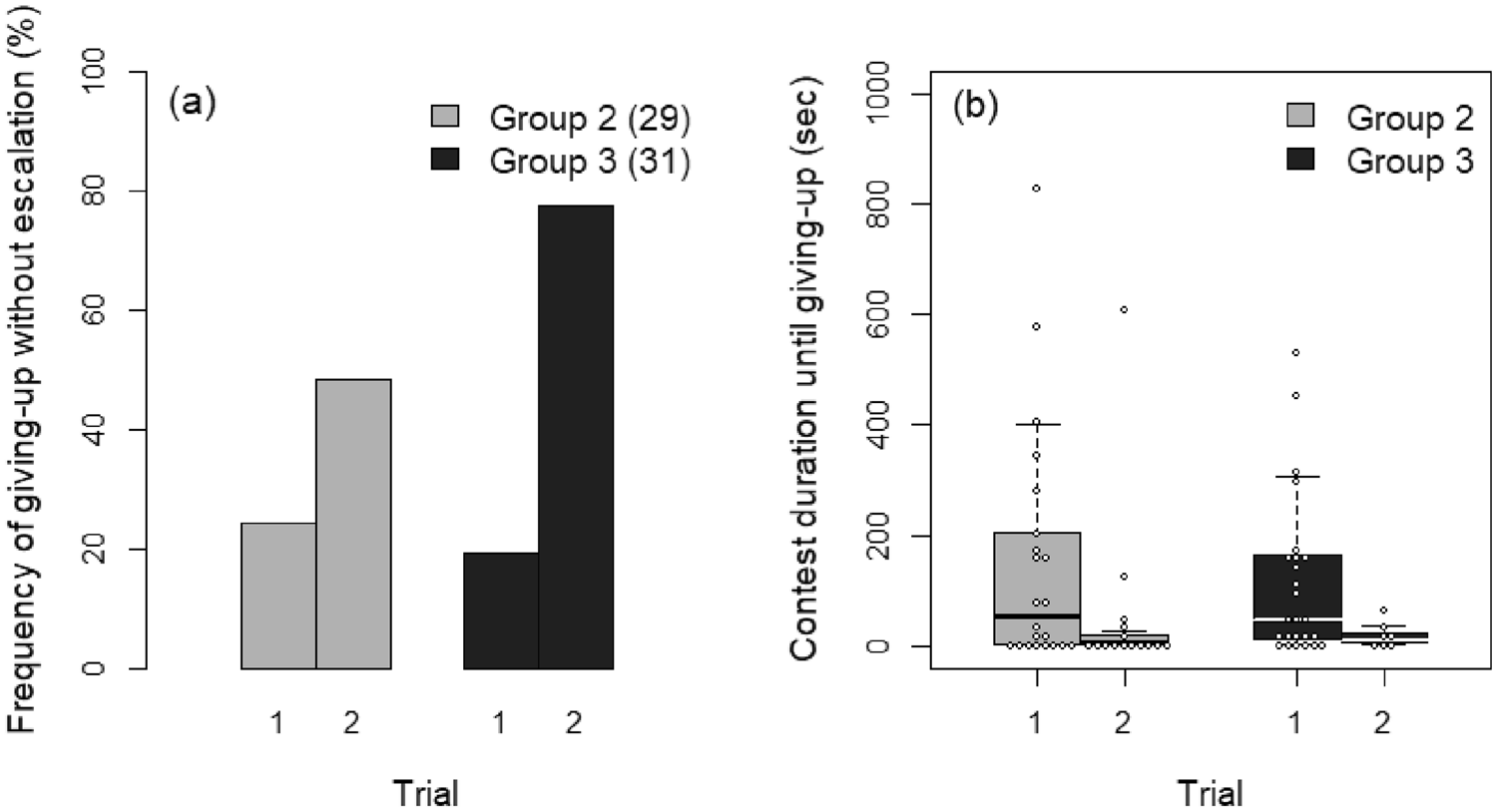
Differences between Groups 2 and 3 in (**a**) the frequency of giving-up without escalation by intruders and (**b**) duration until giving-up by intruders after escalation. Numbers in parentheses in (**a**) indicate sample size. Group 2: opponent that won against a different intruder; Group 3: same opponent as in Trial 1.

In the mixed Cox’s model, Trial × Group interaction was significant (*z* = 2.362, *P* = 0.018). While contest duration until giving-up was similar between trials in Group 1, it was lower in Trial 2 than in Trial 1 in Group 2 (Fig. 3b). However, although Trial 1 was the same for all groups, duration in Trial 1 seemed shorter in Group 1 than in Group 2 (Fig. 3b; see also Fig. 4b for the value of Group 3). The significant Trial × Group interaction might have been caused by this unexpected difference. Smaller intruders gave up significantly sooner after escalation (*z* = − 2.592, *P* = 0.010).

### Established dominance hierarchy

In the comparison between Groups 2 and 3, the Trial × Group interaction was significant in the GLMM (*z* = 2.182, *P* = 0.029). Although the frequency of giving-up without escalation increased in Trial 2 in both groups, the intensity of the trend was greater in Group 3 than in Group 2 (Fig. 4a). DSL_I–G_ also had a significant effect on the decision (*z* = − 2.897, *P* = 0.004).

In the mixed Cox’s model, we excluded the Trial × Group interaction from the model because it was not significant (*z* = − 0.374, *P* = 0.710). In the mixed Cox’s model without the interaction, intruders gave up significantly earlier in Trial 2 than in Trial 1 (*z* = 3.411, *P* < 0.001; Fig. 4b) and with decreasing DSL_I–G_ (*z* = − 2.826, *P* = 0.005). No difference was found between groups (*z* = 0.037, *P* = 0.970).

## Discussion

We conducted random-sized male–male contests of the hermit crab *P. minutus* to examine whether smaller solitary intruders give up contests earlier on the basis of four types of information: size relative to a larger guarding opponent, previous defeats, opponent’s previous wins, and the established dominance hierarchy. We found that three factors increased the frequency of giving-up without escalation: smaller size (Trial 1; Fig. 1a), a previous defeat (Trial 1 vs. Trial 2 in Group 1; Fig. 2a), and re-encountering the same dominant opponent (Group 2 vs. Group 3 in Trial 2; Fig. 4a). The opponent’s previous wins, on the other hand, appeared to contribute to a shorter duration after escalation (Group 1 vs. Group 2 in Trial 2; Fig. 3b). Our previous studies have also shown that the motivation of *P. minutus* intruders to fight is decreased when they encounter a larger opponent in the randomly-chosen contests^29,30^ and had experienced a recent defeat in the similar-sized contests^31^. This is the first study to show that *Pagurus* hermit crab intruders can use all four types of information to decide whether to give up male–male contests and to confirm that the intruders use information about their recent defeats regardless of the experimental method.

The decreased aggression in losers is referred to as the loser effect^19,20^. Many animals show a loser effect when competing against naïve opponents^14,37–39^, and one explanation of this is that losers decrease their self-assessment of RHP relative to others^18,20^. Although Yasuda and Koga^30^ suggest that *P. minutus* assess their RHP relative to their opponent before and after escalation in male–male contests, the greater giving-up frequency in Trial 2 of Group 1 indicates that they also assess their own RHP before escalation, at least after losing. Since intruders’ persistence after escalation did not change even after losing, the loser effect might be based on a perceived but not actual RHP^19^. Although fighting behavior, such as rapping in shell fights of hermit crabs^40^, can carry a cost of depletion of energy reserves^41^, contest duration in Trial 1 did not affect giving-up decision in Trial 2 of Group 1 (Supplementary Fig. S1), and few *Pagurus* males are injured during male–male contests^22,23^. Thus, one contest might not affect actual RHP, via depletion of energy reserves, in our context.

Whereas intruders of *P. middendorffii* ignore a past defeat in their next contest^28^, intruders of *P. minutus* used this information to decide whether to avoid a contest. Yasuda and Koga^30^ have pointed out the possibility that *P. minutus* is more sensitive to fighting costs than *P. middendorffii*; they suggest that *P. minutus* may have more potential mating opportunities than *P. middendorffii*^30^, because *P. minutus* has a longer reproductive period than *P. middendorffii* and only *P. minutus* has multiple oviposition^32,42,43^. Animals showing the loser effect avoid even smaller opponents^38,44^, suggesting that it might be so important for losers to minimize fighting costs that they would not engage even in a fight with a high probability of winning. The loser effect in *P. minutus* might also contribute to cost avoidance and support cost sensitivity in this species.

If *P. minutus* losers maintain their actual RHP after a contest, our analysis (Group 1 vs. Group 2) suggests that intruders of this species can gain information about an opponent’s previous wins after escalation. Because we found no difference in giving-up frequency before escalation in these groups (Fig. 3a), a loser’s decision to give up might be independent of the opponent’s prior wins in the pre-escalation phase. After escalation, however, a significant Trial × Group interaction was detected, and intruders retreated sooner from prior winners than from naïve opponents (Fig. 3b). One possible explanation is that losers could detect an opponent’s cues of a prior win, as can other crustaceans^45^ even after escalation, but why *P. minutus* use this information only after escalation, rather than before escalation like *P. middendorffii*^28^, remains to be explored. Another possible explanation is that the opponent’s actual RHP was increased by a prior win via improved motivation^19^ or fighting skill^46^. On the other hand, since contest duration in Trial 1 was shorter for Group 1 than for the other two groups, this interaction might also be caused by the unexpected value. To determine the relative importance of an opponent’s cues indicating a prior win and an opponent’s enhanced RHP for losers’ decisions, further direct investigation (i.e., random-sized contests between naïve intruders and guarding males with a prior win) is needed.

Greater giving-up frequency in Trial 2 of Group 3 than of Group 2 (Fig. 4a) suggests that *P. minutus* intruders used the previously established dominance hierarchy to avoid familiar, dominant opponents. *Pagurus minutus* could therefore be the second species of *Pagurus* hermit crabs that shows familiar recognition in male–male contests, after *P. middendorffi*^28^. As above, interspecific differences in the loser effect suggest that males of the two species might have different sensitivities to the cost of fighting for a potential mating opportunity. Nevertheless, since intruders of both species avoid escalation against familiar dominant individuals, the established hierarchy should provide reliable information about subsequent decreased chance of success. Since opponent recognition has also been reported in other contexts in *Pagurus* species (*P. bernhardus*^47^ and *P. longicarpus*^48^), this cognitive ability might be widely shared in this group.

Recent studies indicate that information use is flexible within and among species. For example, contestants rely on different information about RHP based on contest phases^23,49,50^, their own positioning^51^, and prior contest experience^17^. In *P. minutus*, intruders use mainly mutual-assessment of RHP to make a giving-up decision but also re-assess their own RHP after losing; information about an opponent’s prior wins might affect different contest phases based on the familiarity of the opponent. *Pagurus minutus* therefore provides an additional example of flexibility in information use. Information use can also vary among closely related species according to interspecific differences in sociality^52^, habitat choice and response to predation^53^, and reproductive characteristics^30,54^. Social cognition is an active field of study but investigations of invertebrates are limited^55^; therefore, investigations in taxonomically diverse organisms are required.

### Supplementary Information

Supplementary Information.

## Data Availability

The dataset is available from the corresponding authors upon request.
