# Supplementary material for: Male Pagurus minutus hermit crabs use multiple types of information in decisions to give up male–male contests
Source: Sci Rep. 2023 Nov 24;13:20654. doi: 10.1038/s41598-023-47947-3 (PMC10673833; doi:10.1038/s41598-023-47947-3)
Supplement: Supplementary file 1 — Supplementary Information. [file 41598_2023_47947_MOESM1_ESM.docx]

Supplementary materials

Male *Pagurus minutus* hermit crabs use multiple types of information in decisions to give up male–male contests

Chiaki I. Yasuda*, Tsunenori Koga

(CIY) Graduate School of Fisheries Sciences, Hokkaido University, Minato-cho, Hakodate, Hokkaido 041-8611, Japan

(TK) Faculty of Education, Wakayama University, Sakaedani, Wakayama 640-8510, Japan

Corresponding author* (CIY) chiaki.y.0210@gmail.com; (CIY) +81-138-40-5548

Fig. S1

There was no significant effect of contest duration in Trial 1 on decision to give up without escalation in Trial 2 in Group 1 (GLM, *N* = 30, *z* = 0.437, *P* = 0.662).


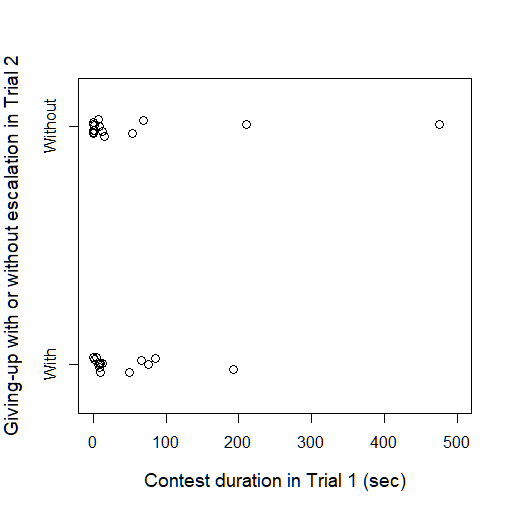


Table S1

Factors affecting intruders’ giving-up decisions, including that with or without escalation or after escalation indicated by a contest duration. For analysis of Trial 1 (a), the generalized linear model (GLM) or Cox’s proportional hazard mode was used. For comparison of two contests (b–d), generalized linear mixed models (GLMMs) or mixed-effect Cox’s models were used.

|  |  |  | Estimate | SE | *z* | *P* |
| --- | --- | --- | --- | --- | --- | --- |
| (a) Trial 1 | | | | | | |
|  | GLM with a binomial error distribution (*N* = 90) | | | | | |
|  |  | Intercept | –2.760 | 0.575 | –4.801 | **<0.001** |
|  |  | DSL_I–G_ | –2.357 | 0.729 | –3.234 | **0.001** |
|  |  |  |  |  |  |  |
|  | Cox’s proportional hazard model (*N* = 70) | | | | | |
|  |  | DSL_I–G_ | –1.487 | 0.433 | –3.435 | **<0.001** |
|  |  |  |  |  |  |  |
| (b) Trial 1 vs. Trial 2 in Group 1 | | | | | |  |
|  | GLMM with a binomial error distribution (*N* = 60) | | | | | |
|  |  | Intercept | –5.551 | 2.247 | –2.471 | **0.014** |
|  |  | Trial 1 vs. Trial 2 | 2.277 | 1.006 | 2.264 | **0.024** |
|  |  | DSL_I–G_ | –2.489 | 1.476 | –1.687 | 0.092 |
|  |  |  |  |  |  |  |
|  | Mixed-effect Cox’s model (*N* = 37) | | | | |  |
|  |  | Trial 1 vs. Trial 2 | –0.231 | 0.413 | –0.560 | 0.576 |
|  |  | DSL_I–G_ | –1.048 | 0.805 | –1.302 | 0.193 |
|  |  |  |  |  |  |  |
|  |  |  |  |  |  |  |
| (c) Group 1 vs. Group 2 | | | | |  |  |
|  | GLMM with a binomial error distribution (*N* = 118) | | | | |  |
|  |  | Intercept | –4.937 | 1.486 | –3.323 | **<0.001** |
|  |  | Trial 1 vs. Trial 2 | 1.860 | 0.636 | 2.925 | **0.003** |
|  |  | Group 1 vs. Group 2 | –0.257 | 0.625 | –0.412 | 0.680 |
|  |  | DSL_I–G_ | –2.668 | 1.062 | –2.512 | **0.012** |
|  |  |  |  |  |  |  |
|  | Mixed-effect Cox’s model (*N* = 74) | | | | | |
|  |  | Trial 1 vs. Trial 2 | –0.189 | 0.387 | –0.489 | 0.620 |
|  |  | Group1 vs. Group 2 | –1.764 | 0.818 | –2.156 | **0.031** |
|  |  | DSL_I–G_ | –1.320 | 0.509 | –2.592 | **0.010** |
|  |  | Trial × Group | 1.301 | 0.551 | 2.362 | **0.018** |
|  |  |  |  |  |  |  |
| (d) Group 2 vs. Group 3 | | | | | |  |
|  | GLMM with a binomial error distribution (*N* = 120) | | | | |  |
|  |  | Intercept | –4.106 | 1.441 | –2.849 | **0.004** |
|  |  | Trial 1 vs. Trial 2 | 1.311 | 0.700 | 1.872 | 0.061 |
|  |  | Group 2 vs. Group 3 | –2.983 | 1.693 | –1.762 | 0.078 |
|  |  | DSL_I–G_ | –2.519 | 0.870 | –2.897 | **0.004** |
|  |  | Trial × Group | 2.305 | 1.056 | 2.182 | **0.029** |
|  |  |  |  |  |  |  |
|  | Mixed-effect Cox’s model (*N* = 69) | | | | |  |
|  |  | Trial 1 vs. Trial 2 | 1.111 | 0.326 | 3.411 | **<0.001** |
|  |  | Group 2 vs. Group 3 | 0.013 | 0.340 | 0.037 | 0.970 |
|  |  | DSL_I–G_ | –1.641 | 0.580 | –2.826 | **0.005** |

Bold values indicate *P* < 0.05.

The ID of intruders was treated as a random effect in all models, and DSL_I–G_ indicates difference in shield length (SL, an index of body size) between intruders and guards.
